# Amniotic membrane for oral surgical defect coverage: A clinical study

**DOI:** 10.6026/973206300223527

**Published:** 2026-06-30

**Authors:** Meenakshi Kothari, Satishkumar G. Patil, Ashwin Shah, Udupikrishna Joshi, Chaitanya Kothari

**Affiliations:** 1 Department of Oral and Maxillofacial Surgery, H.K.E's S. Nijalingappa Institute of Dental Sciences and Research Centre, Kalaburagi, Karnataka, India

**Keywords:** Amniotic membrane (AM), vestibuloplasty, cleft alveolus, oral submucous fibrosis

## Abstract

Reconstruction of oral soft-tissue defects remains limited by donor-site morbidity and inconsistent healing with currently available grafts. In this prospective interventional study, we assessed human amniotic membrane (HAM) as a biologically active scaffold in 15 patients (6 cleft alveolus, 2 oral submucous fibrosis, 7 vestibuloplasty; ages 8-65 years). HAM integrated without complications in all cases, with no necrosis, infection, or immunologic rejection; median pain (VAS) fell from 2 on day 1 to 0 by day 7 (p < 0.00001) and swelling resolved by day 7 (p < 0.00001). Epithelialization reached "good" by 1 month (p < 0.00001); mucosal suppleness remained preserved through 6 months and in OSMF cases interincisal opening improved to 35-50 mm. Thus, data shows the HAM as a safe, donor-site-sparing, clinically translatable biomaterial that accelerates healing and improves function in oral soft-tissue reconstruction.

## Background:

Covering oral mucosal defects is essential for restoring function and improving aesthetics. It also helps prevent complications such as infection and bleeding. Graft coverage protects underlying structures and promotes proper wound healing. It further helps maintain important functions such as speech, mastication and swallowing [1]. Wounds covered with grafts generally heal faster than exposed wounds. They also show less wound contracture during healing [2]. Biological and synthetic grafts provide surface coverage and support tissue repair. They promote healing by maintaining a moist wound environment. This environment facilitates epithelialisation and reduces inflammatory responses [3]. Various graft materials are used in Oral and Maxillofacial Surgery. These include autogenous mucosal grafts and allogeneic collagen membranes [4]. These grafts effectively replace lost tissue and provide wound coverage. However, they have several limitations. These include limited availability, donor-site morbidity and difficulty in harvesting. Other concerns are post-operative pain, graft rejection and infection at the recipient site. Mucosal grafts are technique-sensitive, while collagen membranes may have issues related to processing and availability. Therefore, there is a need to identify alternative biological graft materials with fewer limitations [5]. Biological membrane obtained from placenta; the separated layer of amniotic membrane opens new perspectives [6]. The human amniotic membrane serves as a biological scaffold that facilitates cell adhesion, proliferation and differentiation key processes in tissue repair. Its distinctive attributes, such as angiogenic and antiadhesive effects, bacteriostatic activity, wound protection, pain relief, promotion of epithelialization and favorable safety profile, have driven its widespread adoption across contemporary medical applications [7]. Its superior biological and biophysical properties, along with easy availability and low costs for preparation, storage and application, drive its success over other graft materials and support its wide-ranging clinical uses [8]. Studies across multiple specialties demonstrate the success of human amniotic membrane (hAM) in cell therapy and regenerative medicine. Applications span ophthalmology for corneal reconstruction, dermatology for skin regeneration, orthopedics for joint repair, genitourinary surgery for tissue restoration and dentistry for periodontal regeneration, confirming its efficacy and safety across diverse clinical scenarios [9]. Therefore, it is of interest to show that amniotic membrane coverage of oral surgical mucosal or denuded bony areas accelerates healing, evidenced by reduced pain and swelling alongside faster epithelialisation and mucosal suppleness, monitored over a 3-month follow-up period.

## Methods:

## Source of data:

Patients were recruited from the Department of Oral and Maxillofacial Surgery. The study population included patients requiring vestibuloplasty to increase deficient mandibular vestibular sulcus depth for complete denture rehabilitation. Middle-aged patients with unilateral cleft defects were also included. Patients presenting with reduced mouth opening and a history of areca nut chewing were enrolled in the study.

## Collection of data:

The study included 15 patients (12 males, 3 females; ages 8-65 years) with post-surgical soft tissue defects requiring primary closure. These comprised 6 cases of unilateral alveolar cleft defects in mixed dentition, 2 patients with grade 3 oral submucous fibrosis (OSMF) exhibiting limited mouth opening from areca nut chewing and 7 individuals with shallow mandibular vestibular sulcus depth needing vestibuloplasty for complete denture rehabilitation. All selected patients underwent comprehensive clinical, routine blood and radiological evaluations. Procedures adhered to the ethical standards of the Institutional Ethics Committee on human experimentation and the Helsinki Declaration (2003). The planned procedure was explained to each patient, followed by obtaining informed written consent. Patients with systemic diseases, drug allergies, pregnancy, lactation, immune-compromised status, or local infections in the graft area were excluded from the study.

## Surgical procedure:

## Amnion graft preparation:

Fresh amniotic membrane was sourced from healthy, seronegative mothers undergoing cesarean section. The amnion was separated from the chorion and thoroughly cleansed of blood using copious amounts of tap water. Membranes were then immersed in 85% glycerol solution (prepared by mixing 85 ml glycerol with normal saline to 100 ml) in a large glass bottle and stored at room temperature for 24 hours, followed by transfer to fresh 85% glycerol and refrigeration at 4°C until use [10]. Immediately before application, clean membrane sections (6 x 10 cm^2^) were cut and soaked in 400 mL saline containing 1,000,000 IU penicillin at 48°C for up to 24 hours. The prepared graft material was rinsed in saline solution and applied surgically [11].

## Surgical technique: 

## Surgical technique for vestibuloplasty: 

The patient was scrubbed with savlon and 5% aqueous solution of Povidone Iodine and draping was done. Using local anaesthetic solution (2% lignocaine with 1:80,000 adrenalines), bilateral mental nerve block, lingual nerve block and local infiltration was given. An incision was given on the alveolar ridge and a supraperiosteal dissection is made to the depth planned. The mucosa of the lip was undermined to the vermilion border. The mucosal flap reflected was sutured at the planned vestibular depth with the periosteum using 3-0 absorbable sutures. The denuded periosteal surface was cleared off all the muscle and soft tissue attachments. As failure to this will cause the graft to poorly attach to the underlying periosteum resulting in a mobile graft [12]. The surgical site was thoroughly cleaned with 5% aqueous solution of povidone iodine and saline. The amniotic graft material so prepared is transferred over the surgical site with mesenchymal side against the wound and sutured in place with 5-0 absorbable sutures [13]. The surgical splint was placed after lining with soft liner to prevent formation of dead space and secured either with circum-mandibular suturing or with bone screws of 1.5 X 6mm. The patient was discharged with antibiotics and analgesic coverage for 5 days.

## Surgical technique for oral submucous fibrosis [14]:

The patients were anesthetized using fibre-optic naso-tracheal intubation.

## Resection of fibrous bands: 

The incisions were made with an electrosurgical knife along each side of the buccal mucosa at the level of the occlusal plane away from the Stenson's orifice. Incisions were extended posteriorly to the pterygo-mandibular raphe and anteriorly as far as the corner of the mouth, depending upon the location of the fibrotic bands which restricted mouth opening. These fibrotic bands were incised or excised as detectable by palpation. The wounds created were further freed by manipulation until no restrictions were felt. The mouth was then forced open with a mouth opener to an acceptable range of approximately 35 mm [15].

## Coronoidectomy and muscle myotomy:

The coronoid processes were approached from the wounds created and resected if a 35-mm mouth opening could not be achieved. The insertion of temporal muscle, medial pterygoid and the originating fibres of masseter muscles are relieved [16].

## Removal of third molar teeth:

All the third molars teeth were extracted. A mouth opening of 35 - 50 mm as measured from the incisor edges was considered to be the minimum acceptable opening in an adult.

## Securing the amniotic membrane:

The amniotic membrane was then secured in place with interrupted sutures. The membrane was then secured with Bismuth iodine paraffin paste (BIPP) soaked gauze along with a prefabricated splint using transcutaneous sutures with 1-0 mersilk. The patients were given with IV antibiotics for a period of 5 days followed by oral for another 2 days. They were put on Ryle's tube feeding for a period of 10 days. After 10 days post-operatively the splint was removed. Patients were started on mouth opening exercises 3rd day post-operatively.

## Surgical technique for cleft alveolus repair [17]:

Patients were anaesthetized, scrubbed and draped under Orotracheal General Anaesthesia. Following a crestal incision at the level of gingival sulcus, dissections were made in the scar tissue to reach the bony surface of the cleft walls. The tissue was elevated beneath the periosteum plane to reach the bony surface of the cleft walls. The tissue was elevated beneath the periosteum plane to the levels of the anterior nasal spine anteriorly, the lateral piriform rim superiorly and to the alveolar ridges inferiorly. The nasal mucosa is sutured to avoid any oronasal communication and a bed is prepared. A layer of amnion membrane is placed on the nasal side and autogenous cancellous iliac graft is shaped and placed into the alveolar defect. Another layer of amnion membrane is placed over the graft to prevent fibrosis and stabilize the graft. The flap was subsequently closed in tension free manner. The patients were given with IV antibiotics for a period of 5 days followed by oral for another 2 days. Through irrigation with povidone iodine 5% saline and Chlorhexidine Gluconate 0.2% w/v was done twice a day for the post-operative period of about 15 days [18].

## Evaluation:

The clinical parameters evaluated were divided into 2 groups: subjective (pain) and objective (swelling, epithelialisation and mucosal suppleness). Mouth opening is evaluated in Oral submucous fibrosis cases. Pain was recorded using visual analogue scale (VAS). We divided the VAS scale into four levels, as per the criteria of Az *et al.* [19] as none (0) = 0, Mild (1-3) = 1, Moderate (4-6) = 2 and Severe (7-10) = 3. It was recorded on post-operative 1^st^, 3^rd^, 7^th^ and 14^th^ day post- operatively.Swelling was recorded by visualizing the swelling clinically and evaluated by using the scoring criteria of Siddique *et al.* [20] none (absent) = 0, Mild (just visible and palpable) = 1, Moderate (obvious) = 2 and Severe = 3. Assessment of epithelisation was done at 1^st^ week, 2^nd^ weeks, 1^st^ month and 3^rd^ month's post- operative. It was recorded using the scoring criteria of Rodigruez *et al.* [21] poor (inadequate) = 1, Fair (nearly the entire wound) = 2, Good (entire wound) = 3. Assessment of mucosal suppleness was done at 1 month, 3 months and 6 months post-operative and was recorded using a scoring system: Poor (contracture) = 1, Fair (slightly altered) = 2, Good (similar on both sides) = 3. The interincisal distance was chosen as one of the objective parameter in Oral Submucous fibrosis cases to identify the response to treatment after surgical intervention. Patients were evaluated for mouth opening both intraoperatively and at intervals of 10 days, 1 month, 3 months and 6 months post operatively.

## Results:

This study was intended to evaluate the pain, swelling, epithelialization and mucosal suppleness after the surgical procedures of vestibuloplasty, OSMF and alveolar cleft grafting in oral and maxillofacial region with amnion being used as a graft to cover the surgical defects and the graft beneath it. Categorical data were summarized in proportions and percentages (%) and quantitative (discrete) data were summarized as mean ± SD. The following statistics were calculated in the present analysis: Median, Quartile deviation of assessment of pain, swelling, suppleness and epithelisation and p value by Kruskal-Walli's test. A comparative p-value is calculated with p-value by Post-hoc Dunn's test. P-value by Post-hoc Dunn's test for parameter pain between 1st Day and 3rd Day is 1.0 which is not significant but 1st Day and 7th Day, 1st Day and 14th Day, 3rd Day and 14th Day is less than 0.00001 and 3rd Day and 7th Day is 0.0032 which shows significant results (Table 1). P-value by Post-hoc Dunn's test for swelling parameter between 1st Day and 7th Day, 1st Day and 14th Day is <0.00001 showing significant results (Table 2). p value by Post HOC Dunn's test for parameter epithelisation between 1stWeek and 1st Month, 1st Week and 3rd Month, 2nd Week and 3rd Month is <0.00001 showing significance of the parameter (Table 3). P-value by Post HOC Dunn's test for parameter mucosal suppleness between 1st Month and 3rd Month, 1st Month and 6th Month is >0.0897 showing non-significant results (Table 4). Median pain scores showed a sustained post-operative decline, remaining at 2 on days 1 and 3 and decreasing to 0 by days 7 and 14, indicating complete resolution of pain by the end of the first post-operative week (Figure 1 - see PDF). Median swelling scores decreased from 3 on day 1 to 1 on day 3, with complete resolution by day 7 and persistence of this improvement through day 14, demonstrating effective control of post-operative edema (Figure 2 - see PDF). Epithelialization progressed in a stepwise manner, with median scores increasing from 1 at week 1 to 2 at week 2 and reaching complete epithelialization by 1 month, which remained stable at 3 months (Figure 3 - see PDF). Mucosal suppleness attained a median score of 3 by 1 month and was maintained at 3 and 6 months, reflecting favourable tissue maturation and preservation of functional pliability (Figure 4 - see PDF). Collectively, these findings indicate rapid healing, sustained tissue stability and favourable post-operative functional recovery.

## Discussion:

Delayed and suboptimal wound healing remains a persistent challenge in oral and maxillofacial surgery. This has led to increasing interest in biologically active materials that enhance tissue regeneration and reduce post-operative morbidity. Human amniotic membrane (hAM) has emerged as a promising biomaterial with regenerative and wound-modulating properties [23]. hAM functions as a natural biological scaffold and contains extracellular matrix components that support tissue repair [24]. It also harbors undifferentiated stem cells with multipotent differentiation potential [25]. These cells exhibit low immunogenicity and favorable regenerative capacity [26]. In addition, hAM demonstrates immunomodulatory, immune-privileged and antimicrobial properties [27]. These combined characteristics contribute to enhanced wound stability and improved healing outcomes. Evidence from systematic reviews and randomized controlled trials supports the efficacy of hAM in improving wound healing outcomes [23, 24]. Preclinical and clinical studies further confirm its regenerative potential across multiple tissue types [28]. Epiblastic amniotic-derived cells, formed prior to gastrulation, provide a stable stem cell source.

These cells have demonstrated clinical utility in intraoral reconstruction, corneal repair, tendon regeneration and microvascular reconstruction [28]. Additional applications include burn wounds, cerebral ischemia and neurological disorders. Collectively, these findings support the translational relevance of hAM in regenerative medicine and oral surgery. The present prospective interventional study evaluated the clinical performance of hAM in oral surgical defects. The study included defects associated with oral submucous fibrosis (OSMF), vestibuloplasty sites and alveolar cleft reconstruction. Outcomes assessed included pain, swelling, epithelialization and mucosal suppleness. These parameters were selected to evaluate both inflammatory response and functional tissue regeneration. Pain reduction was significant from post-operative day 3 onward (p = 0.00001), with continued improvement through day 7. Swelling decreased from moderate-severe on day 1 to mild by day 3 and was nearly absent by day 7. These findings indicate effective control of the acute inflammatory phase and accelerated early healing. Epithelialization progressed from fair at 2 weeks to good at 1 month. No post- operative infections were observed. Complete graft adherence was consistently achieved, indicating stable integration of the membrane with the recipient site. Mucosal suppleness was evaluated as a marker of functional healing and scar contracture prevention. This is particularly relevant in OSMF, vestibuloplasty and cleft-related defects where fibrosis can compromise oral function. Most patients demonstrated good mucosal suppleness by 2 weeks, suggesting reduced fibrotic response and improved tissue pliability. Overall, hAM application resulted in reduced inflammation, accelerated epithelialization and improved functional outcomes. These effects are clinically relevant in oral surgical sites, where healing is often compromised by microbial exposure, mechanical stress and tissue deficiency. The findings of this study are consistent with Khademi *et al.,* who reported favorable post-operative pain control during the first post-operative week following hAM application [29]. Similarly, Dawiec *et al.* (2024) demonstrated improved hemostasis, enhanced epithelialization and favorable tissue compatibility with hAM [22]. They also reported reduced post-operative discomfort and improved healing quality. Previous reports have documented the successful use of hAM in oronasal fistula closure and nasal septal perforation repair. Its application as an adjunct in cleft palate surgery has also been described [30]. These findings further support its role in complex maxillofacial reconstruction. This study has limitations. The sample size was small and no control group was included. These factors limit comparative statistical inference. However, consistent improvement across all clinical parameters was observed. This supports the biological effectiveness of hAM in oral wound healing. Future studies should include larger sample sizes and randomized controlled designs. Standardization of application protocols will further strengthen evidence and facilitate wider clinical adoption.

## Conclusion:

Amniotic membrane exhibited substantial regenerative potential, functioning as a biologically active scaffold that enhanced epithelialization, preserved mucosal pliability, minimized post-operative morbidity and prevented graft exposure. Data support its emerging role as a versatile biomaterial for oral soft tissue reconstruction. These present study provide a foundation for future controlled clinical studies.

## Advancement to knowledge:

The present study shows that hAM use leads to reduced early post-operative pain and swelling, faster epithelialization within one month and sustained mucosal suppleness on follow-up. It evaluates three challenging conditions vestibuloplasty, OSMF and cleft alveolar defects expanding evidence beyond isolated defect reports. Findings support hAM as a readily available biological dressing that avoids donor-site morbidity and promotes favorable wound healing, with consistent graft adherence and no infection. Overall, the study provides comparative clinical evidence across multiple oral reconstructive procedures and reinforces the translational value of hAM in maxillofacial regeneration.

## Funding:

No grants or financial support were received from the institution or any commercial source.

## Ethical Approval and Consent to participate:

The research protocol was approved by the Research Ethics Committee of HKE Society's S. Nijalingappa Institute of Dental Science & Research, Kalaburagi (Approval No. HKES/SNDCG/2022-23/1272). Informed consent was obtained from all participants included in the study.

## List of abbreviations:

## Abbreviations & Definition:

hAM - Human Amniotic membrane

OSMF - Oral Submucous fibrosis

## Source of support:

None

## Conflicting Interest (If present, give more details):

None

## Figures and Tables

**Table 1:** Median, Quartile deviation (QD) of Pain and p value by Kruskal-Walli's test

	**1stDay**	**3rd Day**	**7th Day**	**14th Day**
Median	2	2	0	0
QR	0	0.5	0.5	0
P value	1.799e-09(S)			

**Table 2:** Median, Quartile deviation (QD) of Swelling and p value by Kruskal-Walli's test

	**1st Day**	**3rd Day**	**7th Day**	**14th Day**
Median	3	1	0	0
QR	0.5	0.5	0	0
P value	9.334e-12(S)			

**Table 3:** Median, Quartile deviation (QD) of Epithelialisation and p value by Kruskal-Walli's test

	**1st Week**	**2nd Week**	**1st Month**	**3rd Month**
Median	1	2	3	3
QR	0	0	0	0
P value	2.709e-12(S)			

**Table 4:** Median, Quartile deviation (QD) of Assessment of mucosal suppleness and p value by Kruskal-Walli's test

	**1st Month**	**3rd Month**	**6th Month**
Median	3	3	3
QR	0	0	0
P value	0.0432(S)		
